# Interaction of sex and cannabis in adult *in vivo* brain imaging studies: A systematic review

**DOI:** 10.1177/23982128211073431

**Published:** 2022-01-19

**Authors:** Ashley M. Francis, Jenna N. Bissonnette, Sarah E. MacNeil, Candice E. Crocker, Philip G. Tibbo, Derek J. Fisher

**Affiliations:** 1 Department of Psychology, Saint Mary’s University, Halifax, NS, Canada; 2 Department of Psychiatry, Dalhousie University, Halifax, NS, Canada; 3 Department of Psychology, Mount Saint Vincent University, Halifax, NS, Canada; 4 Department of Diagnostic Radiology, Dalhousie University, Halifax, NS, Canada

**Keywords:** Cannabis use, biological sex, sex-based research, sex differences, MRI, fMRI, electroencephalography, magnetic resonance spectroscopy, position emission tomography

## Abstract

Cannabis has been shown to cause structural and functional neurocognitive changes in heavy users. Cannabis use initiation aligns with brain development trajectories; therefore, it is imperative that the potential neurological implications of cannabis use are understood. Males and females reach neurodevelopmental milestones at different rates making it necessary to consider biological sex in all cannabis and brain-based research. Through use of a systamatic review in accordance with PRISMA guidelines, we aimed to understand the interaction between biological sex and cannabis use on brain-based markers. In total, 18 articles containing a sex-based analysis of cannabis users were identified. While the majority of studies (*n* = 11) reported no sex by cannabis use interactions on brain-based markers, those that reported findings (*n* = 8) suggest females may be more susceptible to cannabis’ neurotoxic effects. Unfortunately, a large portion of the literature was excluded due to no sex-based analysis. In addition, studies that reported no sex differences often contained a reduced number of females which may result in some studies being underpowered for sex-based analyses, making it difficult to draw firm conclusions. Suggestions to improve cannabis and sex-based reseach are proposed.

Cannabis is a term frequently used to refer to any consumable product obtained from the *Cannabaceae* family, which consists of three main species: *Cannabis sativa, indica and ruderalis* (Sherma and Rabel, 2019). Cannabis contains more than a 100 cannabinoids and other constituents (Sherma and Rabel, 2019), with the primary psychoactive ingredient being delta-9-tetrahydrocannabinol (THC). Among the other cannibinoids, cannabidiol (CBD), and cannabidiolic acid are thought to counteract some of these psychoactive effects (Bhattacharyya et al., 2010; Iseger and Bossong, 2015; Niesink and van Laar, 2013). The legalisation of medicinal and recreational cannabis has increased in various jurisdictions worldwide with usage rates reported to be on the rise (Government of Canada, 2020; Wadieh et al., 2017). While usage rates may be increasing from 1982 to present, it appears to date that the mean age of first use (age 14 years) has not changed (Červený et al., 2017; Chen et al., 2017; Fleming et al., 1982; Statistics Canada, 2016).

The weight of the current evidence suggests that cannabis use, specifically daily use, affects the cognitive functioning of users (Gorey et al., 2019; Lorenzetti et al., 2019). Many studies report differential effects on males and females; this may be due in part to sex-based differences in brain development occurring around the age of cannabis initiation (age 14 years; Crane et al., 2013b; Ketcherside et al., 2016; Melynyte et al., 2017; Noorbakhsh et al., 2020). Longitudinal data have shown that subcortical structures and grey matter mature and change at different times across adolescence depending on sex (Herting et al., 2019; Lenroot et al., 2007). These developmental processes have been documented to take place between the ages of 8 and 22 years, suggesting significant overlap with the typical onset of cannabis use (14–18 years; Červený et al., 2017; Chen et al., 2017; Government of Canada, 2020). Adding to the complexity of research in this field, some studies report no behavioual changes but significant functional connectivity differences between cannabis users and non-users (Hatchard et al., 2020; Nusbaum et al., 2017), suggesting that cannabis users may recruit additional cortical pathways and brain regions to perform everyday tasks. It has also been shown that these alterations in connectivity are not consistent across sexes (Noorbakhsh et al., 2020). Given that there are sex differences in cannabis use and neurodevelopment, it is possible that one sex may be more susceptible to the impact of cannabis and therefore may have more alterations in cogntive functioning as a result of usage, as has been recently demonstrated in animal models (see Farquhar et al., 2019; Ruiz et al., 2021, for recent work on this topic).

A variety of techniques have been used to examine the potential changes in neurological function and structural changes in cannabis users. Within the realm of magnetic resonance imaging (MRI) and positron emission tomography (PET), studies have showed increases in grey matter in medial temporal clusters (e.g. amygdala, hippocampus, striatum and left prefrontal cortex) in addition to increases in the lingual gyri, posterior cinguelate and cerebellum (Orr et al., 2019) and basal ganglia (Moreno-Alcázar et al., 2018). Decreases in hippocampal and amygdala volumes were also shown in cannabis users (Yücel et al., 2008), in addition to an overall decrease in cerebral blood flow (CBF) in chronic cannabis users, specifically in areas of the prefrontal cortex (for review, see Ogunbiyi et al., 2020). Changes in cortical processing have also been documented with elecroencephalography (EEG) research where cannabis users have been shown to have reduced amplitudes, longer latencies (Ilan et al., 2005; Maij et al., 2017) and worse gating ability (Broyd et al., 2013) to tasks measuring inhibtiory pathways in the brain.

While prior research suggests male and female cannabis users may differ in neural networks (Noorbakhsh et al., 2020) and brain development trajectories (Herting et al., 2019), the vast majority of the literature fails to directly compare male and female users (vs non-users). Therefore, it still remains unclear if recreational cannabis use is neurotoxic (Adam et al., 2020; Pillay et al., 2008; Rocchetti et al., 2013) or neuroprotective (Alexander, 2016; Appendino et al., 2011; Campos et al., 2016; Fernández-Ruiz et al., 2011; Hartsel et al., 2016; Izzo et al., 2009) to males and females and what neural networks (if any) may be impacted with cannabis use. Given the recent increase in cannabis research, it is imperative a timely comprehensive review on exisiting sex, cannabis use and neuroimaging research needs to be completed. The goal of the current review is to expand upon prior reviews (Crane et al., 2013a; Ketcherside et al., 2016) to gain a better understanding of the interaction between cannabis use and sex on brain-based markers of neural functioning through a systematic review of the recent relevant literature.

## Method

### Search strategy

**Figure 1.**
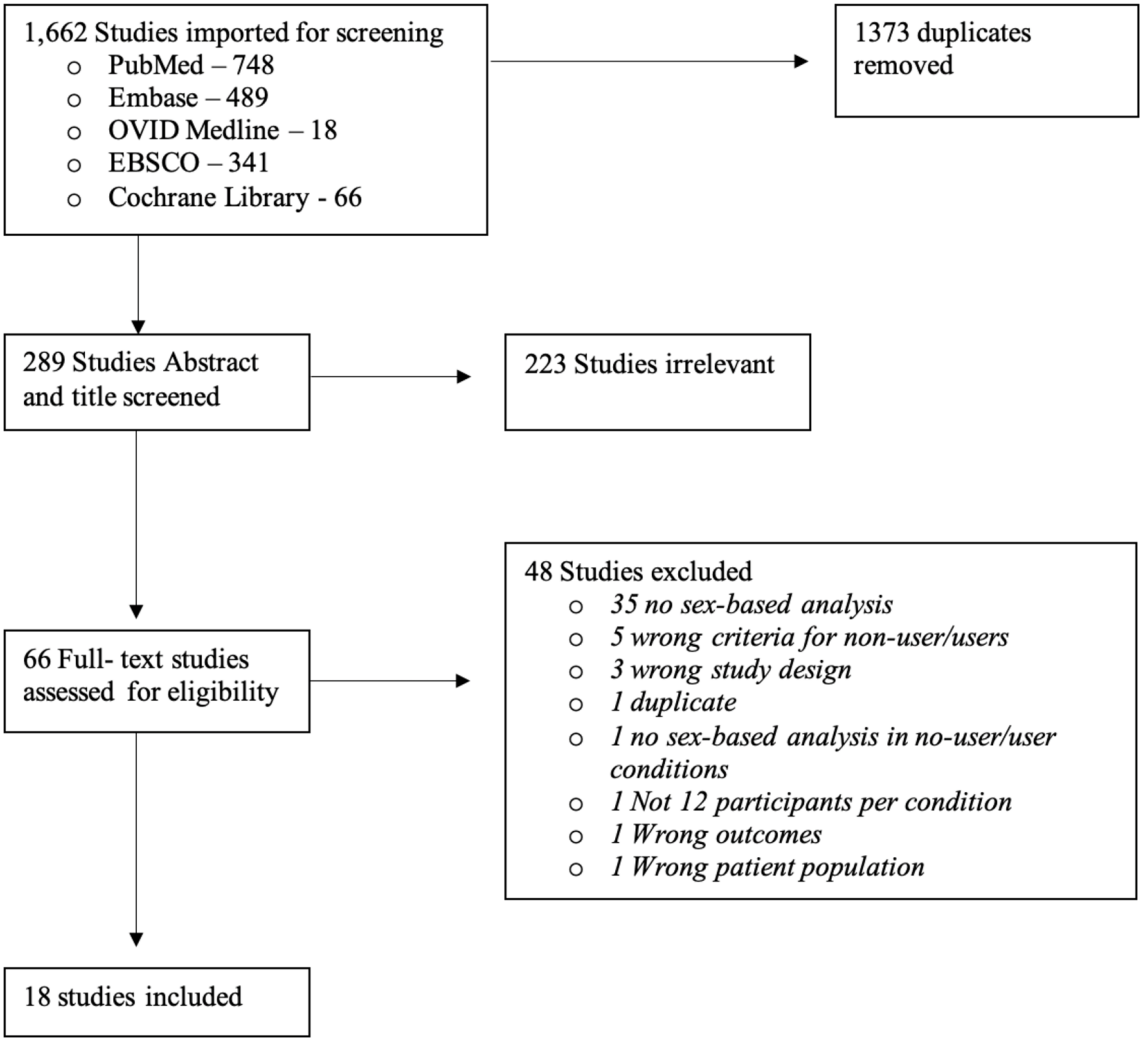
PRISMA diagram. PRISMA: Preferred Reporting Items for Systamatic Reviews and Meta-Analyses (PRISMA) diagram representing the number of studies accepted and excluded at each stage of screening procedures. In addition to this, reasons for why studies were excluded are described here.

A literature review was conducted according to the best practices for conducting systematic literature reviews (Cooper et al., 2018; Siddaway et al., 2018) in accordance with the Preferred Reporting Items for Systamatic Reviews and Meta-Analyses (PRISMA) guidlines (Moher et al., 2009). A comprehensive literature search of PubMed, OVID Medline, Embase, EBSCO and Cochrane libraries was performed to isolate studies using the various search terms relating to sex, cannabis use and neuroimaging (e.g. MRI, PET and EEG; for a full list of search terms, see Table 1). For a full breakdown of how many papers were yielded by each search, see Figure 1.

**Table 1.** Search terms used for systematic review.

Search terms	Key words used
Cannabis	Canna*; Marijuana*; Tetrahydro*; THC
Sex	Gender*; Sex
Brain imaging	Brain imaging; neuroimaging; MRI; fMRI; PET; EEG; ERP

THC: tetrahydrocannabinol; MRI: magnetic resonance imaging; fMRI: functional magnetic resonance imaging; PET: positron emission tomography; EEG: elecroencephalography, ERP: event-related potentials.

Following the removal of duplicates, 289 studies were eligible for title and abstract screening. Titles and abstracts were screened for relevance independently by three reviewers (A.M.F., J.N.B., and S.E.M.) to determine if they met criteria for full text review based on strict inclusion/exclusion criteria; any conflicts were resolved by consensus with an additional member of the review team (D.J.F.). In total, 66 studies underwent the full text review process by the three reviewers. Following the full text review, 18 articles were identified and included in this review. Forward and backward referencing of all articles that reached the full text screen was completed by the first author (A.M.F.): 23 papers were identified, however none of these papers met our inclusion criteria.

### Primary outcome measure

The primary outcome of this systematic review was to assess the interaction of sex and cannabis use on neurological functioning as measured by cognitive testing and neuroimaging studies.

### Inclusion/exclusion criteria

To be eligible for this systematic review, peer-reviewed articles had to meet the following criteria: (1) include a cannabis using condition with comparison group of non-users, (2) contain a sex-based statistical analysis of male and female cannabis users compared with male and female non-users, and (3) contain an analysis using neuroimaging and/or ERP/EEG techniques and report sex-based findings. In each study, cannabis users had to be defined as using cannabis at least 10 or more times in their lifetime and non-users had to be defined as having used cannabis less than 10 times in their life. Studies with less than 12 participants per condition (cannabis users and non-users) were excluded to abide by the latest practices in high-impact neuroimaging research (Szucs and Ioannidis, 2020). In addition, studies were excluded if they involved non-human subjects or full English text was unavailable. Review papers, textbook chapters and unpublished data were not included. Studies published prior to 1990 were excluded.

Despite having vastly different definitions, the current literature uses sex and gender terms interchangeably to describe analysis comparing males and females. To ensure we captured the full depth of the current literature, we used both sex and gender as search terms. Any papers that included an analysis between males and females were included in this review. In addition to this, the current body of literature uses many different names to describe the consumable products obtained from species of the *Cannabaceae* family. As long as the product was harvested/obtained from the *Cannabaceae* family of plants, we included it in our review.

### Data extraction

The data pertinent to our review were independently extracted by the first author. Participants’ characteristics were recorded: total participant number enrolled in the study, sex distribution and mean age. Study characteristics are as follows: classification criteria used of cannabis user and non-user, methodology used (EEG and neuroimaging related) and key findings. Details regarding neuroimaging and EEG methodology and specific characteristics such as model type, magnet strength, stimuli type, event-related potentials (ERPs) of interest and any specific parameters used were included in the data extraction (see Table 2).

**Table 2.** Full description of the studies included in this review.

Author	Participants *n* (female)		Mean age (years)		Classification of cannabis user vs non-user	Neuroimaging method used	Key findings
	Cannabis user	Non-user	Cannabis user	Non-user			
Blest-Hopley et al. (2019)	22 (9)	21 (9)	25.05	24.24	*Cannabis user*: Consumption ⩾ 4 days a week for 2 years; *Non-user*: Used cannabis < 10 times in their lifetime	MRI acquired on a general electric (Milwaukee, Wisconsin) SIGNA HD × 3.0 Tesla system. H-MRS spectra (PRESS – Point RESolved Spectroscopy; TE = 30 ms, TR = 3000 ms; 96 averages). 20 × 20 × 15 mm voxel positioned in the left hippocampus.	*Main effects*: Hippocampal metabolite levels: No main effect of group (*p* = .68) No main effect of sex (*p* = .14) Hippocampal myoinositol levels: Cannabis users < non-users (*p* = .012) Females < Males (*p* = .008) Hippocampal NAA levels: No main effect of group (*p* = .10) No main effect of sex (*p* = .84) *Interaction effects*: Hippocampal metabolite levels: No sex **×** group interaction Hippocampal myoinositol levels: no sex × group interaction (*p* = .89) Hippocampal NAA levels: No interaction between group × sex
Chye et al. (2017)	140	121	28.03	26.12	*Cannabis user*: Mini-neuropsychiatry international interview was used in the Amsterdam population. Severity of dependence scale was used for Barcelona and Melbourne populations. For Amsterdam populations a cut-off of 3 and above was considered a cannabis dependant participant while the Barcelona and Melbourne cut-off was 4 and above to be classified as cannabis dependent. *Non-user*: Scored below the respective cut-offs Cannabis users in Amsterdam used ⩾ 10 days per month or ⩾ 240 days over the past 2 years. Barcelona sample was using 14–28 times a week for a 2-year period, Wollongon was using ⩾ to 3–4 times per week, ⩾ 2 days/month for the last 2 years. *Non-user*: Amsterdam < 50 lifetime uses, no use in past year; Barcelona < 15 lifetime uses, no use in last month; Wollongon no lifetime history of regular use, no use in the past year. Melbourne ⩽ 10 lifetime uses, no use in the past year.	T1-weighted structural MR images varied for the four sites and guidelines were published in prior original research (see Chye et al., 2017, for more information).	Main effects: Lateral orbital frontal cortex volume: No main effect of group (cannabis user vs control) Main effect of dependence (cannabis dependent vs cannabis nondependent) – Cannabis-dep < Cannabis-non-dep (*p* = .029). Medial orbital frontal cortex volume: Main effect of dependence (cannabis dependent vs cannabis nondependent) – Cannabis-dep < Cannabis-non-dep Caudate volumes: No main effect of group Main effect of sex females < males (*p* = .021) No main effect of dependence *Interaction effects*: Lateral orbital frontal cortex volume: Group × hemisphere interaction – left > right; cannabis > control (*p* = .023) Interaction between sex and dependence (cannabis dependent vs cannabis nondependent) – Cannabis-dep female < Cannabis-non-dep female (*p* = .024) Medial orbital frontal cortex volume: No sex × dependence interaction Caudate volumes: Site (Wollongong and Melbourne) × sex interaction: females < males (*p* = .039) Dependence × site (Amsterdam) interaction Cannabis-non-dep Amsterdam > Cannabis – dep Amsterdam (*p* = .047)
Ehlers et al. (2010)	207 (94)	419 (273)	28	31	*Cannabis user: DSM-III-R* criteria for cannabis dependence. *Non-user*: Not-cannabis dependent	EEG – resting data. Six bipolar channels were used (F3-C3, C3-P3, P3-O1, F4-C4, C4-P4, P4-O2). This was all collected using the 10–20 system with impedances below 5 K ohms. Participants were resting during the recording: recording took 10–15 min. Data were filtered using high–low pass filters 1–70 Hz and were free of artefacts.	*Main effects:* No main effect of past month cannabis use on EEG delta activity (controlled for sex and age). *Interactions:* No interactions reported for cannabis use and sex. *Correlations:* Cannabis dependency was correlated with higher delta frequency (1–4 Hz) in the both left and right fronto-central-parietal leads.
Ehlers et al. (2008)	131 (50)	201 (120)	27.5	30.5	*Cannabis user: DSM-III-R* criteria for cannabis dependence. *Non-user*: Not cannabis dependent	EEG-visual paradigm with happy, neutral and sad faces presented for 1000 ms with interstimulus intervals of 1000–1500 ms with a prestimulus interval of 150 ms. A response was needed if the visual was ‘happy or sad’. Seven electrode positions were of interest for the P350 and P450 ERPs of interest (Fz, F3, F4, F7, F8, Cz, Pz), latencies, and amplitudes were averaged across all frontal sites for the P350 and across central parietal sites for P450.	*Main effects:* Main effect of group (controls or other drugs, cannabis dependent and cannabis and other drug dependent) on P350 latency – cannabis dependent group > controls and cannabis and other drugs dependent (*p* < .05) No differences between cannabis dependent and cannabis and other drugs dependent on P350 latency. Main effect of sex on P350 latency – females > males No main effect of P350 amplitude Main effect of cannabis use on P450 latency – cannabis dependency + cannabis and other drug dependency > controls (*p* < .05) Main effect of sex on P450 latency – females > males *Interaction effects:* Sex × group interaction was present with P350 latency, cannabis dependent females > female controls and men with or without cannabis dependence. No interaction between group × sex on P350 amplitude. Females and males did not differ in the control group, but there was an interaction between drug condition and sex on P450 latency – cannabis dependent females > cannabis dependent males.
Filbey et al. (2018)	74(28)	101(51)	31.3	30.3	*Cannabis user*: Lifetime usage ⩾ 5000, and daily use for 60 days preceding testing; *Non-user*: Absence of lifetime use.	MRI data were acquired using a 3 T MR system with a body coil and 8-channel head coil with imaging capability. Time-of-flight angiogram, PC MRI, TRUST MRI and pCASL MRI were used to look at resting brain physiology. T1-weighted image was used as a reference.	*Main effects*: Cerebral blood flow (CBF): No main effect of group Main effect of sex: Females > males (*p* < .001) Oxygen Extraction Fraction (OEF) Main effect of group: Users > non-users (*p* = .03) No main effect of sex Cerebral metabolic rate of oxygen (CMRO_2_) Main effect of group – Users > non-users (*p* = .04) Main effect of sex – Females > males (*p* < .001) *Interaction effects*: Cerebral blood flow (CBF) No group × sex interaction Oxygen Extraction Fraction (OEF) No interaction of sex × group Cerebral metabolic rate of oxygen (CMRO_2_) No group × sex interaction
French et al. (2015)	SYS sample 313 (171); IMAGEN sample: 82 (45)	SYS sample: 636 (319) IMAGEN sample: 251 (143)	Time 1: 14.5; Time 2: 18.5	Time 1: 14.5; Time 2: 18.5	*Cannabis user*: Used cannabis regularly by the age of 16 years; *Non-user*: Had not used cannabis by the age of 16 years.	SYS: T1-weighted images were acquired using a Phillips 1.0 T superconductive magnet. ALSPAC sample: T1-weighted images acquired on a general electric 3.0 T magnet. IMAGEN sample: T1-weighted images acquired on a 3 T scanner from four different manufactures (Siemens, Philips, General Electric, Bruker)	*Main effects*: Saguenay Youth Study Main effect of cannabis use (*p* = .008) No main effect of risk score IMAGEN Study Main effect of cannabis use (*p* = .02) Main effect of risk score (*p* = .007) Main effect of risk score in females (*p* = .007) No main effect of group in females Avon Longitudinal Study of Parents and Children No main effect of group Main effect of risk on cortical thickness – frequent < never users (*p* = .02); frequent users < light users (*p* = .004) *Interaction effects*: Saguenay Youth Study Interaction between group and risk score on cortical thickness in males and females – high-risk cannabis users < non-users (*p* = .002, *p* = .05, respectively) IMAGEN Study Interaction between group and risk score on cortical thickness (*p* = .02) No interaction between group, sex and risk score. Avon Longitudinal Study of Parents and Children No interactions reported. *Correlation* High rank-order correlations between group, cortical thickness and CNR1 in 34 brain regions in low- and high-risk individuals. Largest differences were present between users versus non-users in brain regions with a high density of CNR1 gene expression.
Manza et al. (2018)	30 (8)	30 (10)	29.17	30.23	*Cannabis user: DSM-IV* criteria for cannabis dependence. *Non-user*: Not cannabis dependent.	rsfMRI images were acquired using the Siemens 3T connectome Skyra scanner with a 32-channel coil, T1-weighted and T2-weighted scans were acquired, and rsfMRI was acquired using an echo-planar imaging sequence.	Main effects: Subcortical volume: No main effect of group Trend toward smaller left hippocampal volume cannabis < controls (*p* = .068). Local functional connectivity density (LFCD): Main effect of group – Cannabis > Control in the following areas: Ventral striatum, dorsal midbrain (substantia nigra and ventral tegmental area), brainstem and lateral thalamus *(p* < .0001). No main effect of group on whole-brain functional connectivity analysis based on four clusters. Main effect of age of first use on subcortical LFCD – early initiation > late initiation (*p* = .005). No main effect of sex *Interaction effects:* Local Functional Connectivity Density (LFCD): No group × sex interaction
Maple et al. (2019)	20 (8)	35 (19)	21.7	20.7	*Cannabis user*: Consumption ⩾ 40 times in the past year and at least 50 lifetimes use. *Non-user*: Lifetime uses < 5.	MRI scans were performed using a 3 T scanner (General electric), a T1-weighted, 3-D anatomical brain scan were gathered using a spoiled gradient recalled at stead-state (SPGR) pulse sequence.	*Main effects:* Main effect of past year cannabis use on left rostral anterior cingulate cortex volume: Past year use < no past year use (*p* = .02) No main effect of sex *Interaction effects:* No group × sex interaction
McQueeny et al. (2011)	35 (8)	47 (11)	17.75	18.03	*Cannabis user*: Using cannabis ⩾ 3 years prior to study enrolment, approximately 12 occasions in the past month.	MRI acquisition occurred on a 3 T General Electric Scanner, with 3D T1-weighted spoiled gradient recalled acquisition used to gather high-resolution full brain scans.	*Main effects*: No main effect of group or sex *Interaction effects*: Interaction between group and sex on right amygdala volume (*p* = .03), female users > female controls; male users = male controls
Medina et al. (2009)	16 (4)	16 (6)	18.1	18.1	*Cannabis user*: Consumption ⩾ 60 lifetime uses. *Non-user*: Consumption < 5 lifetime uses.	MRI was acquired by using a 1.5 T General electric Signa LX system with a sagittal acquired inversion recovery prepared T1-weighted 3D spiral fast spin-echo sequence.	*Main effects*: No main effect of group on intercranial volume or prefrontal cortex/intercranial volume. Recent cannabis use on total anterior ventral prefrontal cortex and white matter volumes, recent cannabis use > control (*p* < .05). *Interaction effects*: No group × sex interaction on intercranial volume. Group × sex interaction on prefrontal cortex/intercranial volume (*p* = .09). Prefrontal cortex volumes: Female cannabis users > female controls; Male cannabis users < male controls. *Correlations* Recent cannabis use on total anterior ventral prefrontal cortex and white matter volumes, recent cannabis use > control (*p* < .05). Prefrontal cortex volume was associated with executive functioning in cannabis users, and greater executive functioning was related to smaller total prefrontal cortex volume (*p* < .05). *Multivariate relationships*: Total prefrontal cortex volume predicted executive functioning such that in controls increased prefrontal cortex volume = improved executive functioning; in cannabis users, increased prefrontal cortex volume = decreased executive functioning.
Medina et al. (2010)	16 (4)	16 (6)	18	18	*Cannabis user*: Consumption ⩾ 60 lifetime users, past month use at enrolment. *Non-user*: Consumption < 5 lifetime experiences, none in the past month.	MRI was acquired using a 1.5 T General electric Signa LX system with a sagittal acquired inversion recovery prepared T1-weighted 3D spiral fast spin-echo sequence.	*Main effects*: Cerebellar morphometry: Main effect of sex: right and total cerebellar hemisphere volumes: Females > males. Group status was associated with an increase in posterior inferior vermal volumes: Cannabis users > controls (*p* < .05). Sex predicted anterior, posterior superior and posterior inferior vermis volumes: Female > male (*p* < .02). *Interaction effects*: No group × sex interaction to predict cerebellar volumes Correlations: Smaller anterior, posterior inferior vermis volumes were associated with superior executive functioning.
Roser et al. (2010)	30	30	23	23.9	*Cannabis user*: Regular use ⩾ 3 times per week for a period of at least 2 years. *Non-user*: Absence of lifetime use.	EEG – Auditory paradigm BrainVision (BrainAmp MR, Brain Products GmbH, Munich, Germany) were used to record EEG activity. Data were recorded from 32 Ag/AgCl electrodes arranged in the 10/20 system with a reference of FCz, and ground of FpFz. Eye movement was recorded, and impedances were kept below 5 K ohms.	*Main effects*: Mismatch negativity (MMN) amplitude – frequency deviant: Main effect of cannabis use at Cz. Cannabis users < controls (*p* = .044). Main effect of usage length at frontal sites: long-term users < short-term users (*p* = .014 and *p* = .010, respectively) Main effect of degree of usage (Fz and C4): Heavy users < light users MMN latency: No main effect of group No main effect of subgroup (degree of usage or usage length) No influence of covariates, sex or age for either group. Important points: After nicotine was controlled for, there was no difference between cannabis users and controls on any MMN measures, with the exception of the frequency deviant at site F4 long-term users < short-term users and Fz for heavy < light users – controlling for nicotine usage (*p* < .040 and *p* *<* .020, respectively). *Interaction effects*: No interaction effects reported.
Skosnik et al. (2006)	17 (7)	16 (10)	21.6	23.15	*Cannabis user*: Consumption ⩾ at least once per week. *Non-user*: No history of substance use (including cannabis).	EEG – Participants were sitting related watching photic flickers on a screen with frequencies of 18–25 Hz presented in two separate blocks of 100 trials each 1000 ms each with an interstimulus interval of 1000 ms. Three frontal (F7, F8, Fz), three central (C3, C4, Cz) one parietal (Pz), two temporal (T4, T6) and three occipital (O1, O2, Oz) were used in data collection.	*Main effects:* Steady-state visual evoked potential (SSVEP): Main effect of frequency with both groups having an increased response to 18 Hz stimulation (*p* < .0001) Main effect of sex on SSVEP, females > males (*p* < .008) Phase locking: Main effect of frequency, higher phase locking for 18 Hz (*p* < .0001) Main effect of sex at both 18 Hz and 25 Hz: Females > males (*p* < .007 and *p* < .003, respectively) N160 ERP: Main effect of frequency (*p* < .02) Main effect of group (N160 amplitude): cannabis users < control (*p* < .02) *Interaction effects:* Steady-state visual evoked potential (SSVEP): Group × sex interaction at 18 Hz: females < males *(p* < .047) Phase locking: No group × sex interaction reported N160 ERP: No group × sex interaction reported
Sullivan et al. (2020)	36 (13)	38 (20)	21.4	20.8	*Cannabis user*: Consumption ⩾ 44 times in the past year, at least 100 lifetime uses. *Non-user*: Consumption ⩽ 5 times and no more than 20 lifetime uses.	MRI was acquired using a 3 T Signa LX MRI scanner (General Electric) using a 32-channel head coil. High-resolution images were acquired using a T1-weighted spoiled gradient recalled at steady-state pulse sequence.	*Main effects*: Surface area: Main effect of group in left cuneus: Cannabis > controls (cwp* = .006) Local Gyrification Index: No main effect of group. VO_2_ findings: Increased VO_2_ max was related to greater surface area in the left superior parietal, left inferior parietal, right inferior parietal, and right inferior temporal region in both groups (*cwp* values range from .007 to .0001) Greater VO_2_ max was related to greater local gyrification index in the left superior temporal, right lateral orbitofrontal, right inferior parietal regions for both groups (*cwp* values range from .0015 to .0001). *Interaction effects*: Surface area: Interaction of group × sex in the following areas: left precuneus, left rostral middle frontal, two right superior frontal regions: female users > female controls; male users < male controls. In the right rostral middle region, cannabis users (male and female) < controls (male and female; *cwp* values range from .006 to .002) Local Gyrification Index: Interaction of group **×** sex in the following areas: left precentral and right supramarginal region; female users > female controls; male users < male controls. In the left lateral orbitofrontal, female users < female controls; male users < male controls (*cwp* values range from .0004 to .0001). VO_2_ Findings: Interaction between VO_2_ max and group in the left cuneus surface area. As surface area increased in users, VO_2_ max decreased, opposite was true for controls (*cwp* = .0001). Interaction between VO_2_ max and group in the left lateral occipital region with local gyrification index, as VO_2_ max increased so did local gyrification index for controls, no interaction for users (*cwp* *=* .0004). Three-way interaction of group × sex **×** VO_2_ max was present for local gyrification index in the right supramarginal region (*cwp* = .009). *cwp = cluster wise probability.
Thayer et al. (2020)	105 (33)	41	15.6	15.8	*Cannabis user*: Current user. *Non-user*: Not consuming cannabis.	MRI and DTI were acquired using a 3 T Siemens Trio whole body scanner using 12-channel radio frequency coils. T1-weighted high-resolution images were gathered using a five-echo multi-echo MP-RAGE sequence. DTI scans were gathered using single-shot spin-echo, echo planar imaging with a twice-refocused balanced echo to reduce distortions. A 12-channel radio frequency head coil was used with GRAPPA(×2) and 20 gradient directions.	*Main effects:* Main effect of group on white matter integrity and axonal diffusivity in the left superior longitudinal fasciculus – cannabis use < controls (*p* < .01). *I*nteraction effects*:* No significant group × sex interactions.
Troup et al. (2019)	144 (80)		N/A	N/A	*Cannabis user*: *Heavy users*: Consumption ⩾ once a week. *Casual user*: Consumption ⩽ once a week. *Non-user*: No consumption.	EEG – visual paradigm. Participants sat 30 cm away from the monitor and completed a facial emotion task with full EEG set up using a 25 Ag/AgCl system on a SynAmps2 64-channel QuickCap according to the 10/20 electrode placement with ground midline anterior to Fz and right mastoid as online reference. A 500 Hz sampling rate with bandpass of 0.1–50 Hz and a –200–1000 ms epoch was used. Eye movement was captured. Impedances were kept below 5K ohms.	*Main effects:* P100 amplitude: Males > non-using females for the following emotion conditions: implicit and explicit posterior left region, explicit neutral anterior left and posterior right regions, explicit angry anterior right and posterior left regions, explicit fear posterior left region, empathetic happy anterior right and empathetic fear posterior left regions: non-using (*p* values range from 0.001 to 0.048) Male casual users > Female casual users for the following emotional conditions: Implicit happy and angry, anterior left and right, respectively (*p* = .034; *p* = .002, respectively). Male heavy users > female heavy users for the following emotional conditions: implicit neutral anterior (left and right) and posterior (left and right) regions, happy (anterior and posterior right), angry (anterior right), fear (anterior and posterior left; *p* values range from .49 to .001). Explicit angry anterior left fear anterior right (*p* = .036; *p* = .037, respectively). Empathetic neutral (anterior right and posterior right and left), happy (anterior right and left and posterior left), angry (anterior and posterior right) and fear (anterior and posterior left; *p* values range from .001 to .038). P300 amplitude: No difference of cannabis use in females. Male casual users < male non-users in the empathetic angry condition (*p* = .047) Main effects of group: Non-using males > non-using females in the anterior right and posterior left region for the explicit neutral (*p* = .045, *p* = .035, respectively) and angry (posterior left) conditions (*p* = .044). Casual using males > casual using females in the following conditions: implicit angry (anterior right region; *p* = .017). Heavy using males > heavy using females in the following conditions: empathetic neutral (anterior and posterior right), happy (anterior left; *p* values ranging from .013 to .026). *Interaction effects:* P100 amplitude: Task × Emotion × ROI × Cannabis × Sex interaction (*p* = .028) no difference in female cannabis users. Posterior left region in the explicit angry, fearful emotional condition – Male non-users > Male casual users (*p* = .017). Anterior right region in the empathic angry condition – Male heavy users > Male casual users (*p* = .049). P300 amplitude: Task × Emotion × ROI × Cannabis interaction (*p* = .031) Task × Emotion × ROI × Cannabis × Sex interaction (*p* = .025)
Wiers et al. (2016)	24 (12)	24 (12)	26.8	28.25	*Cannabis user: DSM-IV* criteria for cannabis dependence. *Non-user*: Consumption ⩽ 1 day a month.	PET scans were acquired using a Siemens HR + tomograph. Each participant underwent two PET scans on separate days.	*Main effects*: Baseline brain glucose metabolism: No main effect of group. No main effect of sex. Regional metabolic measures (frontal regions). Cannabis users < healthy controls (*p* < .005). Whole-brain glucose metabolism and methylphenidate (MP)-induced changes: Main effect MP, such that MP versus placebo – whole-brain glucose was higher in healthy controls (*p* = .006), the largest MP-induced increase shown in the following regions: Hippocampus, bilateral thalamus, bilateral occipital cortex, insula and inferior temporal gyrus (*p* < .001). Cannabis users (vs controls) showed decreased response to MP in the following regions: Right putamen, left caudate and midbrain (*p* < .05). *Interaction effects*: Baseline brain glucose metabolism: No group × sex interaction for whole brain Group × sex interaction for regional differences in the bilateral medial frontal gyrus, right superior frontal gyrus and right occipital cortex (*p* < .001), female cannabis users < female controls in the following regions: right anterior cingulate cortex, left superior frontal gyrus and right occipital cortex. Brain glucose metabolism and methylphenidate (MP)-induced changes: Interaction of group × sex × challenge for whole-brain metabolism with MP in female pooled groups, with female controls > female users. A group × sex interaction was found for regional brain effects showing female users < female controls to MP-induced response in the following areas: cerebellum, medial frontal gyrus, pons and hippocampus, thalamus and midbrain (*p* < .01). Correlations: Pooled group means of whole-brain glucose metabolism showed a negative correlation with negative emotional temperament in controls in the following brain regions for controls: fusiform gyrus, inferior parietal cortex, medial and inferior frontal gyrus and anterior cingulate cortex (all *p* values < .005).
Yoon et al. (2006)	High all day = 35 (18); Frequent use = 31 (19)	173 (110)	17.5	17.5	*Cannabis user*: High all day: High for an entire day. *Frequent user*: Consumption ⩾ 1–2 uses per week. *Non-user*: No experience with cannabis.	EEG – Visual paradigm. Data were gathered using three parietal electrodes (P3, Pz, P4). Reference channels were linked earlobes and right shin was ground, eye data were gathered, and impedances were kept below 5 K ohms for scalp electrodes and below 10 K ohms for eye electrodes. All data were acquired through a Grass Model 12A Neurodata system filtered with 0.01 to 30 Hz.	*Main effects*: There was a main effect of group with cannabis users (all levels) < controls on P300 amplitude (*p* < .05). There was a main effect of sex for the P300 amplitude in the ‘any use’ cannabis condition compared with controls: male any cannabis use < female any use (*p* = .034), in addition to this P300 amplitude was significantly impacted for all cannabis use conditions, males < females (*p* values range from .001 to .05). *Interaction effects:* Group × sex interaction between ‘any use’ cannabis users < controls for P300 amplitude, with male cannabis users having reduced amplitudes compared with female cannabis users (*p* = .045). *Correlations:* P300 amplitude was significantly correlated with monozygotic twins suggesting a genetic influence in the P300 amplitude in males and females.

MRI: magnetic resonance imaging; EEG: elecroencephalography; PC MRI: phase contrast MRI; TRUST MRI: T2-relaxation-under-spin-tagging; DTI: Diffusion Tensor Imaging; PET: positron emission tomography, ERP: event-related potentials; NAA: N-acetyl aspartate; H-MRS: proton magnetic resonance spectroscopy; TE: echo time; TR: repetition time; MR: magnetic resonance; SYS: Canadian Saguenay Youth Study; MP-RAGE: magnetization-prepared rapid gradient-echo; ROI: region of interest.

### Risk of bias assessment

**Figure 2.**
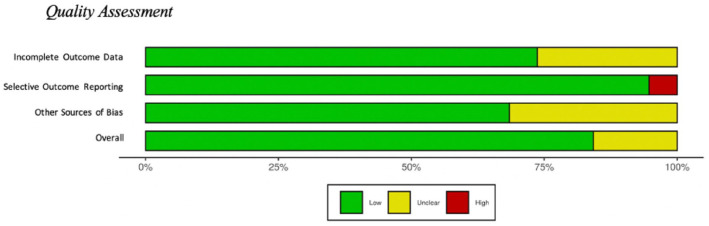
Quality assessment. Quality assessment depicted visually, and this image represents the quality of articles included in the review. This was conducted using the Cochrane Risk of Bias scale. Figure was generated with Robvis.

To evaluate potential systematic risk of bias included in the review, each article was assessed by creating a Cochrane Risk of Bias score for completeness of outcome data, presence of selective outcome reporting, and other sources of possible bias for each article (Higgins et al., 2019; Higgins et al., 2011). Each article was reviewed by two of the first three authors to assess the articles risk of bias providing two scores for each article. Each of the three categories were scored as low risk of bias, high risk of bias, or unclear risk of bias. Overall, there was a low risk of bias for manuscripts examined, see Figure 2 (generated with Robvis; McGuinness and Higgins, 2020).

## Results

The majority of studies (*n* = 13; Blest-Hopley et al., 2019; Chye et al., 2017; Ehlers et al., 2008, 2010; McQueeny et al., 2011; Maple et al., 2019; Medina et al., 2009, 2010; Roser et al., 2010; Skosnik et al., 2006; Sullivan et al., 2020; Wiers et al., 2016; Yoon et al., 2006) report ‘gender’ findings, while *n* = 5 studies (Filbey et al., 2018; French et al., 2015; Manza et al., 2018; Thayer et al., 2020; Troup et al., 2019) report ‘sex’ findings. For consistency in reporting purposes here, all ‘gender’ findings referring to males and females have been changed to ‘sex’ for the remainder of this review as ‘sex’ and ‘gender’ appear to have been used interchangeably in the literature (despite representing distinct constructs), and there was no indication that any participant was transgender. In addition to this, marijuana and cannabis were used interchangeably within the included studies; for consistency, we have chosen to use cannabis when referring to any consumable product obtained from species of the *Cannabaceae* family. Information regarding the polydrug use reported in these studies can be found in Supplemental Material (Table 1).

### MRI, magnetic resonance spectroscopy (MRS) and functional magnetic resonance imaging (fMRI)

In total, 10 studies used structural MRI techniques, while one used MRS and one used fMRI to examine the differences between cannabis users and non-users (males and females).

Three of the 10 MRI studies (McQueeny et al., 2011; Medina et al., 2009; Sullivan et al., 2020) showed that female cannabis users (*n* = 25) versus non-users (*n* = 37) had regional increases in volume of the following cortical areas: left precuneus, left rostral middle frontal region, superior frontal regions and the amygdala. Conversely, male users (*n* = 49) versus non-users (*n* = 52) were found to have global decreases in cortical volume in the same cortical regions: left precuneus, left rostral middle frontal region, superior frontal regions and the amygdala (McQueeny et al., 2011; Medina et al., 2009; Sullivan et al., 2020).

Findings from Chye et al. (2017) also found significant reductions in cortical volume between cannabis users and non-users, specifically around the lateral orbital frontal cortex. Unlike previous work (McQueeny et al., 2011; Medina et al., 2009; Sullivan et al., 2020) that reported an increase in cortical volume in females (*n* = 25), they found that this reduction in cortical volume was most prominent in female cannabis users (*n* = 46; Chye et al., 2017). In addition, they reported no sex × group interaction (Chye et al., 2017). Similarly, Maple et al. (2019) found no sex effects or interactions between cannabis use and sex.

Similar to work done by Chye et al. (2017), McQueeny et al. (2011), Medina et al. (2009) and Sullivan et al. (2020), French et al. (2015) were interested in examining how global and regional cortical thickness varies within young cannabis users (ages of 12–21 years). They found decreased cortical thickness in cannabis users, specifically those with a genetic high risk for schizophrenia (calculated from polygenic risk scores). The authors did not report any overall sex differences between groups (cannabis user vs non-users; French et al., 2015).

To further examine cannabis use patterns and cortical thickness, French et al. (2015) partitioned cannabis use into the following categories: never users (1–2 occasions), moderate users (3–19 occasions) and frequent users (20+ occasions). Participants were then measured to see if cortical thickness changed as a function of usage. Male cannabis users in the moderate and frequent use categories showed differences in cortical thickness compared with never users; however, this was only true for the genetic high-risk condition, and these findings did not hold up in female users (high or low risk) unless age was adjusted. When age was considered, female (high risk) cannabis users (vs non-users) presented with a decrease in cortical thickness. No other sex by group differences or interactions were reported.

Changes in cerebellar volumes have also been reported in this sample, such that marijuana users were reported to have larger posterior inferior vermal volumes, however sex differences or interactions were found in users (Medina et al., 2010). Sex differences were only reported in controls, with increases in right and total cerebellar hemisphere volumes (controlling for intercranial volume (ICV); Medina et al., 2010) observed in female controls.

While the previously discussed studies found differences between cannabis using males and females in terms of brain structure, Thayer et al. (2020) used diffusion tensor imaging (DTI) and voxel-based morphometry (VBM) to measure the association between neural structure and substance use and found no sex differences. DTI was used to visualise whole-brain skeletonised white matter while VBM was used to measured grey matter volume density. While they found cannabis use was associated with inferior white matter integrity (axonal diffusivity in the left superior longitudinal fasciculus [SLF]), no significant sex differences were reported in this sample and no group (cannabis user vs non-user) by sex (male vs female) interactions shown (Thayer et al., 2020).

In addition to the previously reported findings of cortical and cerebellar differences in male and female cannabis users (Chye et al., 2017; French et al., 2015; McQueeny et al., 2011; Medina et al., 2009, 2010; Sullivan et al., 2020), Sullivan et al. (2020) found that female cannabis users showed increases in local gyrification index (LGI) in the left precentral and supramarginal regions compared with non-user females. This indicates that female cannabis users have a larger difference between the ratio of the length of the inner and outer delineation of the sulcus on coronal slices (Sullivan et al., 2020; Zilles et al., 1988). This increase in surface area and LGI was only present in females whereas the opposite was true for males (reduced surface area and LGI; Sullivan et al., 2020).

Finally, one study used MRI techniques such as phase contrast (PC) MRI to measure CBF, to obtain measures of regional blood flow, the pseudo-continuous arterial spin labelling (pCASL) MRI method was used. They were also interested in using T2-relaxation-under-spin-tagging (TRUST) MRI to obtain measures of total brain oxygenation which was used to provide estimates of oxygenation extraction fraction (OEF) and cerebral metabolic rate (CMRO_2_) during periods of rest in cannabis users and non-users (Filbey et al., 2018). Main effects of group (cannabis users vs non-users) were found for OEF and CMRO_2_ such that users had higher OEF and CMRO_2_ values compared with non-users. Sex differences were present for CBF and CMRO_2_ such that females showed higher values than males. Voxel-based analysis indicated cannabis users had significantly higher blood flow in the right pallidum and putamen. There was also a significant effect of sex such that males (vs females) had significantly higher regional CBF in the right insula. Females (vs males) also presented with higher CBF in the left posterior cingulate and bilateral precuneus. No significant interaction between sex and group was found.

Resting state fMRI (rsfMRI) was used in conjunction with local functional connectivity density (IFCD) to observe the influence of cannabis abuse on subcortical functional hub organisation in the brain and their importance to cognitive and mood-related behaviours (Manza et al., 2018). There were no differences between groups (cannabis users and non-users) on measures of cognitive performance using the National Institutes of Health Toolbox measures, part of the Human Connectome Project (e.g. list sorting task, flanker task and pattern completion task). In addition to this, they found no significant differences in the volume of subcortical regions between groups. IFCD analysis revealed significantly higher IFCD in the ventral striatum, dorsal midbrain (substantia nigra and ventral tegmental area), brain stem and lateral thalamus. Higher IFCD was associated with earlier onset of cannabis use; however, they found no effect of sex and no sex by group interactions (Manza et al., 2018).

Blest-Hopley et al. (2019) was the only MRS paper we identified through our search. This group used MRS to determine if chronic cannabis use (*n* = 22) affected levels of glutamate (neurotransmitter affected by neuronal and glial cannabis receptors (Blest-Hopley et al., 2019)), N-acetyl aspartate (NAA; an indicator of neural integrity Ebisu et al., 1994; Moffett et al., 2007) and myoinositol (metabolic marker of neurons and glia) in the brain at central sites with a high density of cannabinoid receptor 1 (CB1). While there were main effects of cannabis use and sex on hippocampal myoinositol levels (decreased levels in cannabis users; decreased levels in females), there was no sex by group interaction.

### Position emission tomography

Position emission tomography (PET) was used in one of the studies found (Wiers et al., 2016) to examine sex differences in cannabis users and non-users. This study examined brain glucose metabolism (an index of neurological functioning) using PET and [^18^F]deoxyglucose (FDG). Cannabis users (vs non-users) showed regional decreases in metabolism in the frontal region, areas of the anterior cingulate cortex, medial and inferior frontal gyrus. Exploratory analysis revealed whole-brain differences between non-users and users such that methylphenidate (MP) increased metabolism in non-users specifically in the midbrain, putamen, caudate, cerebellum and thalamus (Wiers et al., 2016).

Regional differences were found, such that the bilateral medial frontal gyrus, right superior frontal gyrus and right occipital cortex showed a significant interaction between group and sex, with a significant decrease in metabolism in cannabis using females versus female non-users in the following regions: left superior frontal gyrus, right occipital cortex and right anterior cingulate cortex; no significant differences were found for males. While there were no sex by group interactions at baseline, Statistical Parametric Mapping (SPM) analysis revealed a significant sex by group interaction with female cannabis users showing a decreased response to MP in the cerebellum, medial frontal gyrus, pons and in a cluster that includes the following: hippocampus, thalamus and midbrain. They reported males (*n* = 12) showed no differences (Wiers et al., 2016).

### EEG findings

Six studies using EEG to measure brain-based changes in cannabis users (*n* = 462) and non-users (*n* = 839) were identified with our search (Ehlers et al., 2008, 2010; Roser et al., 2010; Skosnik et al., 2006; Troup et al., 2019; Yoon et al., 2006). One study (Troup et al., 2019) examined the effects of cannabis use and sex differences on emotional processing using EEG to capture the visual P100 and P300 amplitudes. Participants were randomly presented with an attentional task with three different conditions: implicit – indicate the sex of the face presented, explicit – indicate the emotion represented and empathic – rate the empathy felt with the emotion displayed.

Males and females differed in both P100 and P300 amplitude, with males reporting enhanced amplitudes for all conditions and heavy using males showing the greatest increase compared to females no other sex by group findings or interactions were reported (Troup et al., 2019). A similar paradigm was used by Ehlers et al. (2008), who used a visual emotion recognition task to assess ERPs in cannabis users with and without other drug dependence. They found an effect of latency, such that there was an increase in P350 and P450 latency for the cannabis using group compared with the control group and the cannabis + other drugs condition, with higher drug dependence being related to longer latencies in the P450. Females exhibited longer P450 latency than males, specifically in the frontal and centro-parietal regions. They also found a drug × sex interaction with P350 and P450 latency that reflected women with cannabis dependency had significantly longer latencies than non-user women and men with and without cannabis dependencies (Ehlers et al., 2008). The authors report that there was no significant effect of P350 amplitude between groups and that there was no sex by drug condition interaction.

Ehlers et al. (2010) were interested in examining the heritability of bipolar EEG spectral phenotypes and genetic risk for substance use disorder, respectively, to understand better their potential relationship with cannabis and alcohol dependence while controlling for several factors. They found no main effect of past month use on EEG delta activity when sex and age were controlled for and no interaction between cannabis use and sex. A correlation was found between cannabis dependency and delta power (1–4 Hz) for both the left and right fronto-central-parietal leads.

Yoon et al. (2006) also found a relationship between cannabis use and ERPs of interest. There was a main effect of group for all cannabis use categories (any use, early use, ‘high’ all day and frequent use), suggesting there is a reduction in P300 amplitude for males compared with females. Furthermore, there was a main effect of sex for the ‘any use’ cannabis condition, with males having significantly reduced P300 amplitudes compared with females (*p* = .002). There was also a sex by group interaction indicating male ‘any use’ cannabis users had significantly reduced P300 amplitude compared with female cannabis users (*p* = .045).

Roser et al. (2010) examined the effect of chronic cannabis use on mismatch negativity (MMN) amplitude and latencies. They found that chronic users versus non-users presented with a reduced MMN amplitude at site Cz for the frequency deviant. No differences in latencies were found among groups or subgroups and there were no effects of covariates such as sex and age (Roser et al., 2010).

Skosnik et al. (2006) was interested in determining if neurophysiological disturbances in the steady-state visual evoked potential (SSVEP) were linked to cannabis use or not. They found a main effect of sex, such that females had a larger SSVEP response than males. A group by sex interaction was also present such that, females had increased SSVEP response compared with males, albeit only at 18 Hz. They reported that individuals who started cannabis use at a younger age had lower SSVEP values to the 18 Hz for both males and females. Finally, there was a main effect of group for the N160 ERP, indicating cannabis use was associated with lower N160 amplitudes; no group by sex interactions were found (Skosnik et al., 2006).

## Discussion

### Global findings

This review assessed the relationship between male and female cannabis users and non-users. We aimed to understand better the interaction between biological sex and cannabis use on brain-based markers. Overall, the majority of studies (*n* = 11) reported no sex by cannabis use interactions on brain structure or functioning as measured by MRI, MRS, fMRI, PET, or EEG (Blest-Hopley et al., 2019; Chye et al., 2017; Filbey et al., 2018; Manza et al., 2018; Maple et al., 2019; Medina et al., 2010; Thayer et al., 2020). This indicates that there may be no differences between male and female cannabis users when measured on neurological functioning.

Overall, in those who did report differences (Ehlers et al., 2008; French et al., 2015; McQueeny et al., 2011; Medina et al., 2009; Skosnik et al., 2006; Sullivan et al., 2020; Troup et al., 2019; Wiers et al., 2016), it appeared that cannabis use impacted female neural functioning more so than males. Across all methodological approaches reviewed, a consistent finding revealed that females experience the potentially detrimental effects of cannabis more than male users. Specifically, we saw deficits in the visual pathway (Troup et al., 2019), decreased metabolism in females in the frontal and occipital region (Wiers et al., 2016) and an overall increase in cerebral volume which was suggested to reflect a deficit in executive functioning (in cannabis users; McQueeny et al., 2011; Medina et al., 2009; Sullivan et al., 2020). All of these findings centre on two major brain regions – the frontal and occipital cortex – suggesting that cannabis may impact these brain regions in females more than other brain regions.

Contradicting evidence was reported regarding how cannabis impacts neural metabolites and CBF (Filbey et al., 2018; Wiers et al., 2016). While Wiers et al. (2016) reported regional decreases in metabolism in users (frontal, anterior cingulate cortex and medial and lateral frontal gyrus), Filbey et al. (2018) reported cannabis users had higher CBF. This is an unexpected finding as usually the two increase and decrease together representing a higher activation of that brain region. The reduced baseline metabolism found in frontal regions in Wiers et al.’s (2016) study has been attributed to an impairment of the frontal baseline metabolism commonly found in cannabis and other drug addictions (Goldstein and Volkow, 2011; Jacobus et al., 2012; Volkow and Fowler, 2000; Volkow et al., 1996). These decreases in metabolism are often related to decreased dopamine D2 receptors found in the striatum (Volkow et al., 1993, 2001, 2013), often related to reductions in self-regulation and higher rates of relapse (Goldstein and Volkow, 2011; Volkow and Morales, 2015). Given this sample (Wiers et al., 2016) had a confirmed diagnosis of cannabis abuse disorder, this explanation helps justify the differences between studies. Filbey et al. (2018) indicated their participants were daily users; however, only for 60 days prior to testing, thus their usage patterns may be different from that of the participants in Wiers et al.’s (2016) study. Given the lower metabolism is often related to relapse (Goldstein and Volkow, 2011; Volkow and Morales, 2015), it would be expected that those with the highest cannabis use would experience these changes. Furthermore, these studies found regional changes in different cortical areas; while Filbey et al. (2018) found changes in the parts of the basial ganglia (pallidum and putamen), Wiers et al. (2016) found changes in the frontal cortex, anterior cingulate and medial inferior frontal gyrus. Therefore, this suggests that cannabis may be impacting these brain regions differently. Future work should aim to understand better this relationship between metabolism and CBF in cannabis users.

### Consideration for interpretation

It is of interest that nine studies (Blest-Hopley et al., 2019; Chye et al., 2017; Ehlers et al., 2008, 2010; Filbey et al., 2018; Manza et al., 2018; Maple et al., 2019; Medina et al., 2010; Thayer et al., 2020) reported null sex by group interaction findings. All but one (Roser et al., 2010) had unmatched sample sizes for males and females in both user and non-user categories. In every scenario, there were fewer females than males and fewer female cannabis users compared with female non-users. This identifies that these findings warrant replication and should be interpreted with caution. To understand better if true sex differences exist, studies need to match sex between conditions to have better powered studies.

Genetic high risk for schizophrenia was shown to moderate the relationship between cortical volume and sex differences in cannabis users such that individuals with a high genetic risk of schizophrenia showed a decrease in cortical volume in males and females; however, individuals without a genetic high risk showed no differences in cortical volume (French et al., 2015). This is an important covariate to consider when discussing cannabis use, as there appears to be a link between early onset of cannabis use and schizophrenia (Arseneault et al., 2002; Fergusson et al., 2003; Henquet et al., 2005; Mauri et al., 2006; Saito et al., 2013; Stefanis et al., 2004; Van Os et al., 2002; Weiser et al., 2002; Zammit et al., 2002). While genetic predispositions remain an important determinant in the development of psychosis/schizophrenia, environmental factors are also at play (e.g. cannabis use, flaws in the glutamate or dopaminergic systems and childhood trauma; Crocker and Tiboo, 2015; Renard et al., 2014; Tibbo et al., 2018). The findings from French et al. (2015) suggest that genetic high risk for schziophrenia may help understand the cortical changes discussed in other studies (McQueeny et al., 2011; Medina et al., 2009; Sullivan et al., 2020) and therefore should be considered and controlled for in future studies.

Only two studies controlled for frequency of use or parsed the sample between heavy and casual users (French et al., 2015; Troup et al., 2019). These studies demonstrated that higher usage or increased frequency of use was related to more substantial changes in cortical volumes (French et al., 2015; Troup et al., 2019). It is possible that the findings reported here are influenced by not controlling for these differences in usage rates. Consumption rates related to the definition of ‘cannabis users’ were different across all studies included in this review, creating variance in our findings and preventing careful comparison between studies. A similar issue is present in how studies address cannabis. Within the review, each study varied on their definition of a cannabis user versus non-user, ranging from a minimum lifetime usage of 5000 times to using cannabis more than once a week to be considered a user. More stringent criteria should be used when recruiting cannabis users and, given the frequency of use findings, subjects should be divided based on frequency of use to ensure we can determine differences between frequent users, casual users and non-users. This will allow for a clear picture of whether casual cannabis use causes neurological changes or if heavy (frequent use) is required to see significant differences between users and non-users. Furthermore, none of the studies included in this review disclosed the type of cannabis and quantity of THC their participants were consuming. This information should be included in future work to understand better the relationship between THC and CBD concentration and alterations in brain structure and function. In addition, with the current rise in THC levels (Chandra et al., 2019), it is more important than ever to fully understand the potential implications of use, without this information it makes drawing cross study conclusions very difficult as we are unsure if the findings are related to THC consumption or CBD (or a mix of both). Moreover, the reporting of polydrug use in participants was not consistent across studies. While some reported all drug use, others reported none. These inconsistencies make interpreting the findings difficult, as we cannot be certain the findings are due to cannabis use and not the polydrug use. Future studies should aim to include measures of polydrug use and measure the impact of these additional drugs on their findings.

Most of the studies included in this review were reporting data on individuals who would be classified as being in emerging adulthood (described as the period between ages 18 and 29 years; Arnett, 2014). This makes understanding the findings difficult, as we cannot be sure any neurological or structural brain changes are due to cannabis use alone and are not simply due to stages of normal development. While all studies had a comparison group of non-users to compare their findings with, this still does not rule out the potential impact normal development may play on these findings.

The majority of studies (*n* = 13; Blest-Hopley et al., 2019; Chye et al., 2017; Ehlers et al., 2008, 2010; McQueeny et al., 2011; Maple et al., 2019; Medina et al., 2009, 2010; Roser et al., 2010; Skosnik et al., 2006; Sullivan et al., 2020; Wiers et al., 2016; Yoon et al., 2006) reported findings in terms of gender, while only six studies (Filbey et al., 2018; French et al., 2015; Manza et al., 2018; Thayer et al., 2020; Troup et al., 2019) reported ‘sex’ findings. While sex has come to be defined for human subjects as their biological sex at birth and is generally divided into two categories – male and female – gender is much more fluid in definition (Rich-Edwards et al., 2018). Gender is typically described by three distinctive components made up of our physical bodies and how we interact with them, our sense of self and how we identify, and our expression of ourselves and our gender (Understanding Gender, 2019) . Therefore, comparing sex and gender may not be possible as they may not be defined the same for all studies and all participants within these studies. Future research interested in examining differences in biological sex should clearly state this and if gender is used, a definition of the construct should be defined for participants and all published documentation.

Finally, a lack of findings due to potentially underpowered studies impacts the quality of research that is being produced, as we cannot be certain that null findings exist. Unfortunately, in cannabis research it is common to fail to include (or document inclusion of) female subjects or have underrepresentation in the female participant groups. This fails to tell the entire story of how cannabis use may impact neurological functioning as we are failing to measure half of the population (females). In addition to this, we know that females neurologically mature at a different rate than males, and the fluctuations in hormone levels may differentially impact females in comparison with males (Galvez-Buccollini et al., 2012; Lenroot et al., 2007). For all these reasons, future research is needed to better examine the true differences in neurological functioning between male and female cannabis users and non-users. Finally, it was hard to draw cross study comparisons between the research included in this review as each study was focused on select regions of interest within the brain. Unfortunately, this makes drawing general conclusions across studies harder as cannabis may be differentially impacting these brain regions. Future work should aim to incorporate a more global analysis of the impact of cannabis use on neural functioning to understand better its impact on the brain.

## Supplemental Material

sj-docx-1-bna-10.1177_23982128211073431 – Supplemental material for Interaction of sex and cannabis in adult in vivo brain imaging studies: A systematic review Click here for additional data file. Supplemental material, sj-docx-1-bna-10.1177_23982128211073431 for Interaction of sex and cannabis in adult in vivo brain imaging studies: A systematic review by Ashley M. Francis, Jenna N. Bissonnette, Sarah E. MacNeil, Candice E. Crocker, Philip G. Tibbo and Derek J. Fisher in Brain and Neuroscience Advances
