# Supplementary material for: Interaction of sex and cannabis in adult in vivo brain imaging studies: A systematic review
Source: Brain Neurosci Adv. 2022 Jan 19;6:23982128211073431. doi: 10.1177/23982128211073431 (PMC8793398; doi:10.1177/23982128211073431)
Supplement: sj-docx-1-bna-10.1177_23982128211073431 – Supplemental material for Interaction of sex and cannabis in adult in vivo brain imaging studies: A systematic review [file sj-docx-1-bna-10.1177_23982128211073431.docx]

| **Figure 1.**  Polydrug use reported in each paper separated by user status | | |
| --- | --- | --- |
|  | **Polydrug use** | |
|  | **Cannabis users** | **Non-users** |
| Blest-Hopley et al. 2019 | Yes – cannabis users were also using the following substances (n,%).  Nicotine: 12 (55%)  Alcohol: 116 (90%) days of alcohol use in the past year  Cocaine: 9 (41%) past year  MDMA: 6 (27%) past year  Hallucinogens: 4 (18%) past year | Yes – non-users were using the following substances (n,%).  Nicotine: 4 (19%)  Alcohol: 86 (96%)  Cocaine: 2 (10%) past year  MDMA: 2 (10%) past year  Hallucinogens: 0 |
| Chye et al. 2017 | Cannabis users : Alcohol on average 24 standard drinks reported per month Tobacco: on average 254.96 cigarettes consumed per month  All averages were calculated across all four testing locations. | Non-users: alcohol use per month 20 standard drinks on average. Tobacco: on average 31 cigarettes smoked per month.  All averages were calculated across all four testing locations. |
| Ehlers et al. 2010 | 60% of participants met criteria for alcohol dependence.  28% had a dual diagnosis | 60% of participants met criteria for alcohol dependence.  28% had a dual diagnosis |
| Ehlers et al. 2008 | 66 participants had marijuana and another drug dependence, of these individuals, 89% had alcohol dependence.  n = 47 had only marijuana dependence, within this group 66% had alcohol dependence. | 33% had alcohol dependence |
| Filbey et al. 2018 | Excluded for history of drug abuse, or regular tobacco use (> 1 pack of cigarettes/month) | Excluded for history of drug abuse, or regular tobacco use (> 1 pack of cigarettes/month) |
| French et al. 2015 | Supplemental material mentioned that participates were asked about their drug use using the National Longitudinal Survey of Children and Youth and the Quebec Longitudinal Study of Child Development, however, does not report the results for this questionnaire in text or in supplemental. | Supplemental material mentioned that participates were asked about their drug use using the National Longitudinal Survey of Children and Youth and the Quebec Longitudinal Study of Child Development, however, does not report the results for this questionnaire in text or in supplemental. |
| Manza et al. 2018 | Groups were matching on alcohol and tobacco use. 36 participants met the DSM-IV criteria for cannabis use disorder, three also met the criteria for comorbid alcohol use disorder and were subsequently excluded from analysis. | Groups were matching on alcohol and tobacco use |
| Maple et al. 2019 | Participants were excluded if they used any form of psychotropic medication, reported using more than 10 cigarettes per day or used heavy drugs more than 25 times in their life (excluding cannabis).  Cannabis users reported (Females: *M* = 339.6, *SD* = 349.9; Males: *M* =433.5, *SD* = 275.7) standard drinks of alcohol in the past year, and using (Females: *M* = 46.4, *SD* = 84.9, Males: *M* = 264.3, *SD* = 448.2) cigarettes in the past year. | Participants were excluded if they used any form of psychotropic medication, reported using more than 10 cigarettes per day or used heavy drugs more than 25 times in their life (excluding cannabis). Non-users reported consuming (Females: *M* = 59.4, *SD* = 102.6; Males: *M* =115.5, *SD* = 187.6) standard drinks of alcohol in the past year, and on average (Female: *M* = .8, *SD* = 2.8; Male: *M* = .1, *SD* = .3) cigarettes per year. |
| McQueeny et al. 2011 | Participants were excluded if they used any form of psychotropic medication, met the criteria for abuse or dependence of substances other than cannabis, and if their use of other illicit drugs exceeded 30 lifetime uses. Users reported on average (Female: *M* = 239.4, *SD* = 211.2; Male: *M* = 185.7, *SD* = 138.9) lifetime uses of alcohol, this usage was significantly higher than that of non-users (*p* < .05). Lifetime use of other drugs was reported as (Female: *M* = 8.1, *SD* = 6.1; Male: *M* = 4.5, *SD* = 7.1) this was significantly more use than what was reported by non-users (*p* < .05). | Participants were excluded if they used any form of psychotropic medication, met the criteria for abuse or dependence of substances other than cannabis, and if their use of other illicit drugs exceeded 30 lifetime uses. Non-users reported on average (Female: *M* = 26.6, *SD* = 61.24; Male: *M* = 21.0, *SD* = 27.5) uses of alcohol this use was significantly lower than that of users (*p* < .05). Lifetime use of other drugs was reported as (Female: *M* = .2, *SD* = .6; Male: *M* = .5, *SD* = 2.2) this was significantly lower than the use reported in cannabis users (*p* < .05). |
| Medina et al. 2009 | Participants were excluded if they used any form of psychotropic medication, or if they tested positive for any substance use during the 28-day abstinence period. Cannabis users had less than 25 lifetime uses of drugs other than cannabis, alcohol and nicotine. Alcohol use was reported in lifetime episodes with females (*M* = 301, *SD* = 132), reporting higher use than males (*M* = 158, *SD* = 124) | Participants were excluded if they used any form of psychotropic medication, or if they tested positive for any substance use during the 28-day abstinence period. No use of other drugs except nicotine and alcohol, were not considered heavy drinkers based on scales used. Alcohol use was reported in lifetime episodes with females (*M* = 32, *SD* = 64), reporting higher use than males (*M* = 17, *SD* = 37) |
| Medina et al. 2010 | Participants were excluded if they used any form of psychotropic medication, or if they tested positive for any substance use during the 28-day abstinence period. Cannabis users had less than 25 lifetime uses of drugs other than cannabis, alcohol and nicotine. Lifetime uses of other drugs (*M* = 7, *SD* = 9). | Participants were excluded if they used any form of psychotropic medication, or if they tested positive for any substance use during the 28-day abstinence period. No use of other drugs except nicotine and alcohol, were not considered heavy drinkers based on scales used. No reported lifetime uses of other drugs. |
| Roser et al. 2009 | All cannabis users also reported cigarette use on average for 5.6 years, using *M* = 8.6 cigarettes per day. No history of other drug use. All participants were screened for amphetamines, ecstasy, benzodiazepines, cannabinoids, cocaine, methadone, and opiates before testing to ensure no illegal drug use prior to testing and to verify cannabis use in users. On average users reported consuming *M* = 4.1 (*SD* = 5.7) alcoholic drinks per week | Nine of 30 participants (non-users) were self-disclosed cigarette smokers on average for 6.2 years with a consumption of 9.9 cigarettes a day. No history of other drug use. All participants were screened for amphetamines, ecstasy, benzodiazepines, cannabinoids, cocaine, methadone, and opiates before testing to ensure no illegal drug use prior to testing and to verify cannabis use in users. Controls reported consuming *M* = 1.9 (*SD* = 1.8) alcoholic drinks per week. |
| Skosnik et al. 2006 | Participants had no other illicit substance use during the past 6 months (excluding cannabis use), additionally, no DSM-IV diagnosis of alcohol dependence. If participants reported consuming more than 2 alcoholic drinks per day (1 for females) they were excluded from the study. Males reported consuming on average *M* = 5.4 (*SD* = 5.4) drinks per week while females reported consuming *M* = 3.6 (*SD* = 4.9). | Participants had no history of illicit substance use and no DSM-IV diagnosis of alcohol dependence. If participants reported consuming more than 2 alcoholic drinks per day (1 for females) they were excluded from the study. Males reported consuming *M* = .4 (*SD* = .8) alcoholic drinks per week, while females reported *M* = 3.6 (*SD* = 4.9). |
| Sullivan et al. 2020 | No current use of any psychoactive medications, no excessive illicit drug use (> 20 uses in their life per drug category). Average alcohol consumption per year was *M* =338.7 (*SD* =300.8) standard drinks. Past year tobacco use was *M* = 214.6 (*SD* = 483.7) cigarettes consumed. | No current use of any psychoactive medications, no excessive illicit drug use (> 20 uses in their life per drug category). Average alcohol consumption per year was reported as *M* = 100.6 (*SD* = 173.6). Past year tobacco use was reported as *M* = .5 (1.97) cigarettes consumed. |
| Thayer et al. 2020 | *n* = 17 participants reported consuming alcohol and cannabis while, *n* = 27 reported using cannabis and tobacco and finally, *n* = 40 reported using all three (alcohol, cannabis, and tobacco). On average participants reported consuming *M* = 1.01 (*SD* = 2.09) alcoholic drinks in the past 30 days and reported consuming *M* = 12.32 (*SD* = 13.59) cigarettes in the past 30 days. | On average participants reported consuming *M* = 1.01 (*SD* = 2.09) alcoholic drinks in the past 30 days and reported consuming *M* = 12.32 (*SD* = 13.59) cigarettes in the past 30 days. |
| Troup et al. 2019 | Reported not testing for another illicit drug use | Reported not testing for another illicit drug use |
| Wiers et al. 2016 | Participants were excluded if they had a history of substance abuse/addiction (other than cannabis abuse/dependence and nicotine abuse/dependence). Participants were excluded if drugs were detected during the drug screen (other than cannabis). The sample contained 10 active cigarette users and two former cigarette users, no significant differences in cigarette use between groups. | Participants were excluded if they had a history of substance abuse/addiction (other than cannabis abuse/dependence and nicotine abuse/dependence). Participants were excluded if drugs were detected during the drug screen (other than cannabis). The sample contained 10 active cigarette users and two former cigarette users, no significant differences in cigarette use between groups. |
| Yoon et al. 2006 | Nine percent of males (*n* = 43) in this sample and 7% of females (*n* = 43) reported frequent (at least 1-2 times/week) use of alcohol with *n* = 50 (15%) males and *n* = 35 (10%) of females reporting ‘binge drinking’ (consuming seven or more drinks/week for an ongoing basis of two or more months. Frequent of cigarette use (30 days in a typical month) was described as *n* = 65, (13%) in males and *n* = 89, (15%) in females. Five percent of males (*n* = 25) and 4% of females ( *n* = 22) reported an extreme use of cigarettes with more than 15 cigarettes consumed on a daily basis. Other drugs were recorded such as amphetamines where 5% of males (*n* = 26) and 5% of females (*n* = 28) reported use in their lifetime, additionally 3% of males (*n* = 16) and 4% of females (*n* = 23) reported use of psychedelics. Finally, there were 7% of males (*n* = 35) and 7% of females (*n* = 41) who used at least four other substances. | Nine percent of males (*n* = 43) in this sample and 7% of females (*n* = 43) reported frequent (at least 1-2 times/week) use of alcohol with *n* = 50 (15%) males and *n* = 35 (10%) of females reporting ‘binge drinking’ (consuming seven or more drinks/week for an ongoing basis of two or more months. Frequent of cigarette use (30 days in a typical month) was described as *n* = 65, (13%) in males and *n* = 89, (15%) in females. Five percent of males (*n* = 25) and 4% of females ( *n* = 22) reported an extreme use of cigarettes with more than 15 cigarettes consumed on a daily basis. Other drugs were recorded such as amphetamines where 5% of males (*n* = 26) and 5% of females (*n* = 28) reported use in their lifetime, additionally 3% of males (*n* = 16) and 4% of females (*n* = 23) reported use of psychedelics. Finally, there were 7% of males (*n* = 35) and 7% of females (*n* = 41) who used at least four other substances. |
